# The Relationship between Depression Symptoms and Severity of Coronary Artery Disease in Patients Undergoing Angiography

**DOI:** 10.18502/ijps.v15i4.4305

**Published:** 2020-10

**Authors:** Arsalan Salari, Asieh Ashouri, Arezoo Javadzadeh Moghtader, Zahra Ahmadnia, Iman Alizadeh

**Affiliations:** Cardiovascular Diseases Research Center, Department of Cardiology, Heshmat Hospital, School of Medicine, Guilan University of Medical Sciences, Rasht, Iran.

**Keywords:** Coronary Angiography, Depression Symptom, Severity of Coronary Artery Disease* Stenosis

## Abstract

**Objective:** Cardiovascular diseases are the main cause of mortality worldwide. Depression is one of the effective factors in the incidence of cardiovascular diseases like coronary artery stenosis. This study aimed to investigate the relationship between depression symptoms and severity of coronary artery disease (CAD) in patients scheduled for angiography.

**Method** **:** This prospective, cross sectional research was conducted on as many as 401 patients scheduled to undergo angiography at Dr. Heshmat heart hospital as the referral center in the north of Iran in 2016. Before cardiac catheterization, patients' demographic information (age, gender, level of education, and place of residence) and patients’ medical history (history of diabetes mellitus, hypertension, and family history of cardiac disease) were obtained. Also, Beck Depression Questionnaire 2 (BDI II) was completed by a psychologist before angiography. After collecting the data, SPSS v.21 and statistical tests such as Spearman correlation, and Mann-Whitney U regression were used to analyze the data.

**Results: **After controlling for age, sex, and having history of diabetes mellitus, no relation was found between having depression symptoms and more frequency of vessel involvement (OR = 1.35, 95% CI: 0.92 to 1.98, P =0.130) or higher severity of CAD (OR = 1.47, 95% CI: 0.95 to 2.28, P = 0.087). The results were similar for the relation between severity of depression symptoms and CAD extent or CAD severity.

**Conclusion: **The results of this study showed that in patients undergoing angiography, depression symptoms were not related to CAD severity and number of involved vessels. Depression was associated with angina, independently of CAD severity. Our study found no significant correlation between CAD severity and severity of depression. The reason may be that measuring depression at a single time point cannot accurately reveal the impact of this problem on the trend of atherosclerosis over time.

Coronary artery disease (CAD) is one of the prevalent problems and the main cause of death worldwide (1, 2). It kills 17 million people per year which accounts for 30% of the total deaths worldwide. It is estimated that this number will rise to 24.8 million people in 2020 if appropriate preventive measures are not taken (3). 

Insufficient blood supply and inability to supply the cardiac muscle with enough oxygen result in coronary artery disease (1). Family history, age, gender, hypertension, diabetes mellitus, smoking, the increase in cholesterol levels, overweight, and physical inactivityare common risk factors in cardiovascular diseases. Studies in recent decades have shown that psychological factors play a similar role in mortality of the patients with coronary artery disease (4). 

Depression is prevalent in patients with coronary artery disease and monitoring its symptoms is highly recommended to patients (5). There are many different hypotheses elaborating on the association between depression and coronary artery disease: Inflammatory cytokines cause progression of the coronary disease by forming plaques on the wall of arteries (6). 

Different studies have shown that in patients with or without history of cardiac diseases, depression is related to increased levels of inflammatory cytokines such as IL6, IL1, and CRP (7). 

Serotonin has a role in depression pathophysiology (8). On the other hand, in the vessels at risk of obstruction, serotonin causes aggregation of platelets through binding to 5-hydroxytryptamine (5-HT) receptors; therefore, a huge amount of serotonin in blood can lead to cardiac ischemia (6, 9). In addition, another noteworthy point is that depression causes endothelial dysfunction. This will contract the arteries in blocked sites and is likely to cause coronary obstruction and myocardial ischemia (10, 11). 

In many studies, depression has been regarded as a risk factor in patients with coronary artery disease (12) and is seen as an independent risk factor in these patients (13). The incidence of depression increases the risk of mortality and recurrent infarction (13-15). Depression at the time of undergoing angiography in patients with coronary artery disease doubles the risk of suffering a cardiac event a year after angiography (16, 17). Identifying depression symptoms in patients with CAD is highly crucial as these patients have poor prognosis (18, 19). Depression exerts much more extensive and important influence on the daily performance of the patients and symptoms of the disease than angiographic findings (20). Therefore, these patients should be screened and receive treatment for depression (21). 

Given the role of depression in the outbreak, process, and prognosis of cardiovascular diseases (22), it seems that depression symptoms are related to severity of CAD and coronary artery disease in patients undergoing angiography. Although most studies have investigated the relation between depression and cardiac disease, little research with inconsistent findings has been conducted on the relation between depression and severity of coronary artery disease. Thus, this study aimed to examine the relation between depression and severity of coronary artery disease.

## Materials and Methods

This prospective, cross sectional study was conducted on patients undergoing elective angiography and hospitalized in the angiography ward of Dr. Heshmat heart hospital, a referral center in the north of Iran. Accessibility sampling method was employed and patients were followed consecutively from October 2016 to November 2016 for 5 months for coronary angiography to investigate stable angina pectoris or severe chest pain that was possibly caused by CAD. A total of 401 patients participated in the present study. Our institutional ethics committee approved the study, and informed consent was obtained from all patients. 

The inclusion criteria were age over 18, willingness to participate in the study, not having verbal and hearing problems, not having sever urgent conditions, complete consciousness at the time of research, and not being mentally handicapped. 

The exclusion criteria were cardiomyopathy, history of congenital heart disease, chronic renal failure, previous myocardial infarction or coronary artery bypass grafting, medical treatment for chronic psychosis, or recent medical treatment for depression, and incomplete study form.

***Instruments***

Two separate questionnaires were used for the purpose of data collection. The first part collected data on demographic characteristics, including age, gender, level of education, and place of residence; and patients’ medical history, eg, history of diabetes mellitus, hypertension, and family history of cardiac disease. The second part was Beck Depression Inventory 2 (BDI II). All forms were completed by a psychologist prior to angiography at the CAD lab. To measure the severity of depression, BDI II, a 21-item questionnaire, was used, and its internal consistency has been calculated to be between 0.73 and 0.92 by Beck et al (23). The range of scores on the inventory is between 0 and 3, with zero indicating absence of depression and 3 the maximum level of depression. The range of the scores is between 0 and 63. The patients were asked to choose an option which expresses their feelings best in the past 2 weeks. 

Evaluation of depression was conducted based on the following points: 0-13: normal; 14-19: minor depression; 20-28: average depression; and scores over 29: severe depression (24). The inventory has been standardized for the Iranian context and the reliability and validity of the instrument has been confirmed (25). 

The psychometric characteristics of the Persian version of BDI-II in the patients with CHD were examined by Ahmadi et al in 2019; and its internal consistency which was measured using Cronbach's alpha was estimated at 0.90. The obtained correlation between BDI-II and PHQ-9 and GAD-7 was 0.74 and 0.65, respectively (p < 0.001) (26).

***Procedure***

To determine the severity of the CAD (the stenosis of the heart’s coronary arteries and the number of arteries involved) angiography, which is a diagnostic procedure, was used. The findings of angiography were interpreted by 2 specialists blindly. In the first step, one of the 2 specialists examined the films visually in the CAD lab; and in the second step, a second specialist examined the films to add the accuracy of the examinations. The findings of angiography were categorized based on the severity of the disease and number of arteries involved. 

Angiograms with no visible atherosclerotic changes in the coronary arteries were considered as normal. Stenosis that reduced the lumen diameter was defined as mild < 50% stenosis, moderate 50% to 70% stenosis, and severe > 70% stenosis.

The presence of stenosis in 1, 2, or 3 of major coronary arteries (left main, right coronary artery, left anterior descending, and circumflex) was respectively considered evidence of single, 2-, or 3-vessel coronary artery disease (27).

To observe ethical considerations, written informed consent was obtained from the participants in the study. The data were collected by a trained research expert supervised by a psychologist. The trained research expert introduced herself and the goal of the study and assured the patients that their information will remain confidential and the results of the study will be used in a research paper. 

The collected data were analyzed using SPSS V.21. Mean (SD) or frequency (percentage) was reported to describe the patients’ characteristics. Normal distribution assumption was met for both age and total cholesterol using the Kolmogorov–Smirnov test. In the univariable analyses, Mann-Whitney U test and Spearmen correlation coefficients were used to assess the relation between patients’ characteristics and depression level, CAD extent and CAD severity, for nominal and ordinal variables, respectively. In the multivariable analyses, for controlling the possible confounding effect of patients’ sex, age, and history of diabetes mellitus, ordinal regression model was used to determine the relation between depression level and CAD extent or severity. Assumption of parallel lines was tested and met. There was no multicollinearity among independent variables. To have a comparable sample size in groups, in the regression analyses, CAD severity was grouped into 3 normal, mild/moderate, and severe categories. All statistical tests were 2-sided and P < 0.05 was considered as statistically significant. 

## Results

Patients’ characteristics are presented in Table 1. Of the total of 401 patients, depression symptoms were observed in 165 (41%) patients; whereas, 92 (23%), 50 (12%), and 23 (6%) of patients had mild, moderate, and severe depression, respectively. Female sex (P < 0.001), lower education (P = 0.002), living in a rural area (P = 0.007), and having history of hypertension (P = 0.030), or heart disease (P = 0.030) were related to higher level of depression.

Frequency of patients with normal, 1-, 2-, 3- or more vessel involvement CAD was 133 (32%), 78 (20%), 84 (21%), and 106 (27%), respectively. Patients with more vessel involvement CAD were older (mean age 56, 58, 60, and 61 years for normal, 1-, 2-, 3- or more vessel involvement CAD groups, respectively; r = 0.23, P < 0.001), were more likely to be male (male ratio of 40%, 56%, 67%, and 62% of patients with normal, 1-, 2-, 3- or more vessel involvement CAD groups, respectively (P<0.001), and were more probable to have a history of diabetes mellitus (DM ratio of 28%, 39%, 43%, and 51% of patients with normal, 1-, 2-, 3- or more vessel involvement CAD groups, respectively; P < 0.001). 

Data showed that severity of CAD and frequency of vessel involvement CAD were highly correlated (r = 0.841, P < 0.001); also, similar results were observed for severity of CAD. Patients with severe CAD were older

(mean age 56, 56, 61, and 60 years for normal, mild, moderate, and severe CAD, respectively; r = .20, P < 0.001), were more likely to be male (male ratio of 40%, 50%, 54%, and 64% of patients with normal, mild, moderate, and severe CAD, respectively; P < 0.001), and were more likely to have a history of diabetes mellitus (ratio of 28%, 29%, 39%, and 47% of patients with normal, mild, moderate, and severe CAD, respectively; P < 0.001).

The results of univariable analysis revealed no relation between depression level and frequency of vessel involvement CAD (r = 0.009, P = 0.864) or severity of CAD (r = -0.011, P = 0.829). Also, in the multivariable analysis, after controlling for sex, age, and history of diabetes mellitus, no statistically significant relation was found between depression level and frequency of vessel involvement or severity of CAD (P >0.1 for all odds ratios).

The odds ratios and 95% confidence intervals are shown in Table 2. Increasing the odds ratios were not linear with regards to higher level of depression symptom. 

However, compared with patients with no depression symptom, patients with severe depression symptoms had higher severity of CAD (OR = 1.88, 95%CI: 1.10 to 3.21, P = 0.020); however, it should be noted that the sample size was small in patients with severe depression level. 

The results of assessing depression symptom variables in 2 levels (patients with and without depression symptoms) were similar. Controlling for sex, age, and history of diabetes mellitus, the odds ratio of having more vessel involvement CAD and higher severity of CAD in the patients with depression symptoms versus patients without depression symptoms was 1.35 (95% CI: 0.92 to 1.98, P =0.130) and 1.47 (95% CI: 0.95 to 2.28, P =0.087), respectively. 

Based on the results in Table 2, controlling for other independent variables in the model, the odds of males having more vessel involvement CAD and higher severity of CAD was 2.87 (95% CI: 1.92 to 4.30) and 3.66 (95% CI: 2.30 to 5.81) times that of females (P < 0.001), respectively. The odds of patients with history of diabetes mellitus having more vessel involvement CAD and higher severity of CAD was 2.54 (95% CI: 1.72 to 3.74) and 2.98 (95% CI: 1.89 to 4.69) times those of patients without diabetes mellitus (P < 0.001), respectively. Also, 1-year increase in age was related to an increase in the odds of having more vessel involvement CAD and higher severity of CAD, with an odds ratio of 1.04 (95% CI: 1.02 to 1.07, P < 0.001) and 1.05 (95% CI: 1.02 to 1.07, P < 0.001), respectively (Table 2).

**Table 1:** Characteristics of the Participants Based on Depression Status

**Factor**	**Total ** **(n=401)**	**Depression status, no. (%)** *				**P value**
		**None** 236(59)	**Mild** 92(23)	**Moderate** 50(12)	**Severe** 23(6)	
Sex, no. (%)						<0.001
male	219(55)	158(72)	41(19)	12(6)	8(4)	
female	182(45)	78(43)	51(28)	38(21)	15(8)	
Age (years), mean (SD)	59 (9.31)	58 (9.56)	59 (9.80)	59 (6.95)	58 (9.50)	0.843
Education, no. (%)						0.002
Illiterate	162(40)	79(49)	46(28)	24(15)	13(8)	
Not holding a high school diploma	171(43)	104(61)	34(20)	23(14)	10(6)	
High school diploma and higher	68(17)	53(78)	12(18)	3(4)	0(0)	
Smoker, nonsmoker (%)						0.537
Yes	87(22)	58(67)	13(15)	9(10)	7(8)	
No	314(78)	178(57)	79(25)	41(13)	16(5)	
Living location, no. (%)						0.007
Urban	226(56)	147(65)	44(20)	26(12)	9(4)	
Rural	175(44)	89(51)	48(27)	24(14)	14(8)	
Hx. HTN, no. (%)						0.030
Yes	183(46)	94(51)	50(27)	28(15)	11(6)	
No	217(54)	141(65)	42(19)	22(10)	12(6)	
Hx. DM, no. (%)						0.095
Yes	157(39)	85(54)	37(24)	25(16)	10(6)	
No	244(61)	151(62)	55(23)	25(10)	13(5)	
Hx. HD (yes), no. (%)						0.030
Yes	188(47)	100(53)	46(25)	30(16)	12(6)	
No	213(53)	136(64)	46(22)	20(9)	11(5)	
TC (mg/dL), mean (SD)	156(40.04)	151(35.69)	162(49.20)	156(37.18)	173(40.80)	0.051
Extent of CAD, no. (%)						0.864
None	133(32)	77(58)	25(19)	22(17)	9(6)	
1-Vessel CAD	78(20)	51(65)	20(26)	4(5)	3(4)	
2-Vessel CAD	84(21)	48(57)	22(26)	10(12)	4(5)	
3 or more-Vessel CAD	106(27)	60(57)	25(24)	14(13)	7(30)	
Severity of CAD, no. (%)						0.829
Normal	133(32)	77(58)	25(19)	22(17)	9(6)	
Mild	14(4)	8(57)	3(21)	2(14)	1(7)	
Moderate	26(7)	20(77)	4(15)	2(8)	0(0)	
Severe	228(57)	131(58)	60(26)	24(11)	13(6)	

SD, Standard Deviation; Hx, History of; HTN, Hypertension; DM, Diabetes; HD, Heart Disease; TC, Total Cholesterol; CAD, Coronary Artery Disease.

* Percent in rows was calculated based on the total number of patients in each level.

**Table 2:** Estimated Regression Coefficients of Independent Variables with CAD Risk

**Factors**	**OR**	**95%CI**		**P value**	**OR**	**95%CI**		**P value**
Age	1.04	1.02	1.07	<0.001	1.05	1.02	1.07	<0.001
Sex (males vs females)	2.87	1.92	4.30	<0.001	3.66	2.30	5.81	<0.001
Hx. DM (yes vs no)	2.54	1.72	3.74	<0.001	2.98	1.89	4.69	<0.001
Depression symptom status								
Mild vs None	1.34	0.60	2.97	0.475	1.30	0.53	3.17	0.570
Moderate vs None	1.23	0.68	2.21	0.496	0.99	0.51	1.89	0.965
Severe vs None	1.40	0.89	2.21	0.142	1.88	1.10	3.21	0.020

Hx indicated history of; DM, Diabetes; CAD, Coronary Artery Disease; OR, Odds Ratio; CI, Confidence Interval.

## Discussion

The results of this study showed that in patients undergoing angiography, depression symptoms were not related to CAD severity and number of involved vessels. Being a female, low education level, living in a village, and a history of hypertension and cardiac diseases were found to be related to high levels of depression. 

The relation between psychological variables like depression and CAD in patients undergoing angiography has been investigated in different studies and conflicting results have been reported. A few studies, however, have examined the relationship between depression and severity of CAD; and their results have been different (22, 28-34). For example, a Cohort study on 164 patients undergoing angiography in Greece showed no significant difference in the score of depression between patients with nonsevere CAD and severe CAD, and only the score of anxiety in males was related to severe CAD. In these cohort studies, the average age of 82% of male participants was 65 ± 11 years. Females in this study had CAD with less severity and more depression (31). Carney et al also found no significant relationship between the severity of CAD in depressed patients and the nondepressed (32). In a study by Hayek et al, 5158 patients undergoing cardiac catheterization were studied. It was found that depression was related to cardiac angina and was independent of CAD severity; depression was also found as the most important predictor of chest pain, which was caused by depression and not the severity of the disease (33).

In contrast to the findings of this study, a study by Vural et al in 2009 showed no association between CAD and depression initially, but after controlling for sex differences and other confounding variables, they found that every 1-point rise in the depression score was associated with an average of 5% to 6% increase in the abnormal coronary angiographic findings or definitive coronary artery disease, respectively (P = 0.01 and P = 0.002) (31). To score depression as we did in the present study, they used BDI, but they checked the results of angiography using Judkins technique.

Also, in a study by Ekici et al, which has been done on 225 patients undergoing elective angiography, the results showed a significant relationship between CAD and the score of depression and anxiety. Although the size of the reported correlation explained the weakness of the relationship, to explain the difference between this research and our study, it can be pointed out that Ekici’s research was a case control study and the severity of CAD was determined by Gensini score. Furthermore, the scoring of depression was estimated by Hospital Anxiety and Depression Scale (HADS) (22). While in our study, the severity of CAD was estimated visually and the scoring of depression was determined by Beck II inventory.

 In a cross sectional study in 2007, a statistically significant difference was reported between the average score of depression in a CAD and Non-CAD group; and hyperactivity of the noradrenergic system was one of the most important mechanisms explaining this relationship (34).

In addition, in the present study, female gender was related to higher levels of depression; and many other studies have reported similar findings (31, 34-37). On the other hand, in studies by Kokkou et al and Hayek et al, no significant relationship was reported between depression and sex (29, 33). However, in our study, based on the angiography findings, the frequency and severity of CAD in males was more than females, which is consistent with many previous studies (22, 34). 

Moreover, in our study, reviewing the history of cardiac disease among the participants showed a significant relationship between depression symptoms and hypertension and cardiac disease history, which corresponds to the results of the study by Hayek in 2017 (33). In addition, Abbasi et al in a study in Tehran Cardiac Center showed that depression had a relationship with hypertension (35). In this study, depression did not have a significant relationship with DM and hypocholesteremia, which is in contrast with the study of Vural et al in 2007 and 2009 (31, 34).

One of the limitations of the present study was its small sample size in different levels of CAD severity. Moreover, in this study, the emphasis was on depression symptoms as self-reported by the patients and without clinical interview. Moreover, patients might have reported their feelings incorrectly in self-reports, and this could adversely affect the findings of the study. 

The presence of a psychologist and heart specialists in the research team, accuracy in diagnosis of CAD severity, arterial involvement based on angiography, and the detailed data collected from patients’ cardiac medical history were the merits of this study. Furthermore, this study was done at a referral hospital in north of Iran, which covers a large number of patients in this area.

## Limitations

As for the limitations of the present research, one could refer to the use of the patients' self-reports for collecting accurate data which influences the findings.

## Conclusion

In general, our study showed no relationship between depression and severity of coronary artery disease in patients undergoing angiography. The reason may be that measuring depression at a single time point (at the time of research and not paying attention to the history of disease) cannot accurately reveal the impact of this problem on the trend of atherosclerosis over time.

Considering the cross sectional nature of our study, which prevents discovering the relationship between depression and severity of coronary artery disease, conducting longitudinal studies with larger sample sizes is highly recommended in the future. Additionally, further studies with better designs are warranted to explore the impact of psychological intervention on CAD severity and its long-term outcome.
